# Single incision laparoscopic cholecystectomy with conventional instruments and ports: Initial experience at tertiary care public sector Hospital

**DOI:** 10.12669/pjms.333.12930

**Published:** 2017

**Authors:** Abdul Razaque Shaikh, Syed Asad Ali, Ambreen Munir, Aijaz Ali Shaikh

**Affiliations:** 1 Abdul Razaque Shaikh, Professor & Dean Surgery Department of Surgery, Liaquat University of Medical &Health Sciences, Jamshoro, Sindh, Pakistan; 2 Syed Asad Ali Associate Professor, Department of Surgery, Liaquat University of Medical &Health Sciences, Jamshoro, Sindh, Pakistan; 3 Ambreen Munir Associate Professor, Department of Surgery, Liaquat University of Medical &Health Sciences, Jamshoro, Sindh, Pakistan; 4 Aijaz Ali Shaikh Assistant Professor, Department of Surgery, Liaquat University of Medical &Health Sciences, Jamshoro, Sindh, Pakistan

**Keywords:** SILC, Conventional Instruments, Safety

## Abstract

**Objective:**

To find out safety and feasibility of single incision laparoscopic cholecystectomy (SLIC) using conventional instruments.

**Methods:**

This study was conducted at surgical department of LUMHS Jamshoro Pakistan from Jan: 2014 to Dec: 2015. All cases of symptomatic cholelithiasis that consented for laparoscopic surgery were included. The exclusion criteria were acute cholecystitis, acute gall stone pancreatitis, common bile duct stones and patients with co-morbid. A midline 3cm incision made supraumbilically and 10mm port placed. Two 5mm ports placed on either side of umbilicus slightly superior and laterally in or-der to triangulate. A 2/0 prolene suture placed through the infundibulum of the gall bladder to achieve retraction. The rest of the procedure is like standard 4 ports laparoscopic cholecystectomy.

**Results:**

Total no of cases were 50. The age ranged from 30-59 years (mean 35.20 years ±4.886.) There were 43(86%) females and 07(14%) males. The mean operating time was 80 minutes (range 50-120 ±16.020). Four (8%) cases were converted to standard four ports laparoscopic cholecystectomy due to bleeding and difficult dissection in Calot’s triangle. Minimal blood loss was observed during the procedure with no postoperative complications. The range of hospital stay was 1-2 days (mean 1.08 ±0.274).

**Conclusion:**

SILC is a safe and feasible procedure with conventional laparoscopic instruments without additional cost of single port and articulated instruments. The cosmetic results are excellent with minimal increase in the operating time.

## INTRODUCTION

Minimal invasive surgical techniques have replaced open surgical procedures with the advantage that it has reduced the trauma of access. Today laparoscopic cholecystectomy when performed is considered as the “gold standard procedure for cholelithiasis”.^1^ The reason is very simple; just to ligate the cystic artery and cystic duct; in open cholecystectomy not only wide skin incision is needed but all layers of the abdominal cavity have to be incised and therefore trauma of access to the area of gall bladder is much more than the trauma of the required procedure. This is probable explanation of the world wide popularity of the laparoscopic cholecystectomy.

At present conventional laparoscopic cholecystectomy has significantly reduced trauma of access and is being performed using four ports and therefore needs, though small, but four incisions. During recent past search continues to reduce access trauma even more and as far as history of single incision laparoscopic surgery concern, it dates back to 1992, when single puncture laparoscopic appendectomy was performed.^2^ In 1997 laparoscopic cholecystectomy was performed with two trans-umbilical.^3^ Consequently, efforts of biomedical engineers were aimed to develop instrument that requires minimum access trauma but has convenience of many port assembled in a single instrument.^4^ The efforts proved fruitful and finally single incision laparoscopic surgery (SILS) using single port articulated instrument came into practice.^5^

In some center of the world, cholecystectomy is being done by SILS & NOTES (Natural Orifice Trans luminal Endoscopic Surgery) and results claimed are superior to that of the conventional four port cholecystectomy.^6^ The results of several randomized clinical trials have shown that single port, single incision cholecystectomy is a safe procedure with postoperative outcomes similar to those of conventional laparoscopic cholecystectomy.^7^ However, the cost of the instrument is a major limiting factor for it wide acceptance and therefore presently practiced in only stat of art centers of the world. Rationale of the current study was to further reduce the trauma of access while performing laparoscopic cholecystectomy, without increasing cost of the procedure. Trauma of access reduced further by giving single incision, while using conventional instrument used for four port cholecystectomies the cost of the procedure will not increase. Therefore, the objective of current study was to find out safety and feasibility of single incision laparoscopic cholecystectomy (SLIC) using conventional instruments.

## METHODS

This prospective study was conducted at Surgical Department of Liaquat University of Medical & Health Sciences Jamshoro, from January 2014 to December 2015. The patients of either gender having symptomatic gall stone disease with BMI of less than 35 and, who have consented for laparoscopic surgery were included in the study. Patients having acute cholecystitis, acute gall stone pancreatitis, common bile duct stones and with co-morbidities were excluded. Patients were provided information sheet elaborating the rationale and detail of the procedure. They were also informed about the possibility of more than one incision and conversion to open method as safety of the patient is of prime importance. Patient information sheet was in their native language without medical terms. All patients were assured that surgery will be performed by senior laparoscopic surgeon having clinical and practical experience of laparoscopic surgery of at least 10 years.

Outcome measures studied includes; operative time, intra-operative and post-operative complications, post-operative pain, hospital stay and patient acceptance of the aesthetic results. Visual analogue scale (VAS) used to record the post-operative pain, while aesthetic acceptance was recorded on a Likert scale from 1-5; where one for complete satisfaction and five for total dissatisfied. For aesthetic results patient were followed for 12 weeks and then results were recorded once.

### Surgical procedure

All steps of surgical procedure were identical to that used for standard laparoscopic cholecystectomy. “The patient was placed in supine position and the operating table was tilted in an anti-Trendelenburg position, rotated to the left”. Both the surgeon and the camera assistant were standing on the left side of patient with the monitor on the opposite side. A midline supra-umbilical incision was made, and a 10-mm port was inserted. After creating a pneumo-peritoneum of 12 mm Hg, peritoneal cavity was explored and feasibility of the laparoscopic procedure was evaluated. The other two five mm ports were placed on either side of umbilicus slightly superior and lateral in order to obtain a triangle with the 10-mm trocar at the apex. All instruments used were same as use for four ports laparoscopic cholecystectomy. A 2/0 PROLENE suture was placed through the infundibulum of the gall bladder. The rest of the procedure is like standard laparoscopic cholecystectomy. The technical challenges faced and overcome during the procedure include triangulation, retraction and exposure, inline vision and instrument crowding. All cases were video recorded for the easy reference of all those interested in the procedure.

## RESULTS

Total no of cases in our study were n=50. The age of the patients ranged from 30-59 years (mean 35.20 years±4.886). There were 43(86%) females and 07(14%) males. (Table-I) The operative time ranges from 50-120 with mean of 80 ±16.020 minutes. Four (8%) cases were converted to standard four incisions laparoscopic cholecystectomy due to bleeding and difficult dissection in calot’s triangle. Minimal blood loss was observed during the procedure with no postoperative complication. The median pain scale was three. The range of hospital stay was 1-2 days (mean 1.08 ±0.274). At follow-up, no late complications were observed, and cosmetic results as far as the scar is concerned were satisfactory (Table-II).

**Table-I:** Patient Characteristics (n=50).

***Gender***	
Female	43 (86%)
Male	07(14%)
***Age (years)***	
Mean	35.20 ±4.886
Range	30-59
Co-morbidities	nil
Conversion	4 (8%)
Post-Operative Complications	Nil

**Table-II:** Intraoperative and Postoperative results.

	*Mean*	*SD±*	*5% trimmed Mean*	*Median*	*Interquartile range*
Mean operating time (min)	80	16.020	79.71	80	24
Mean Pain Scale (VAS)	3	1.106	2.955	3	1
Length of Hospital stay (days)	1.08	0.274	1.03	1	0

**Fig.1:**
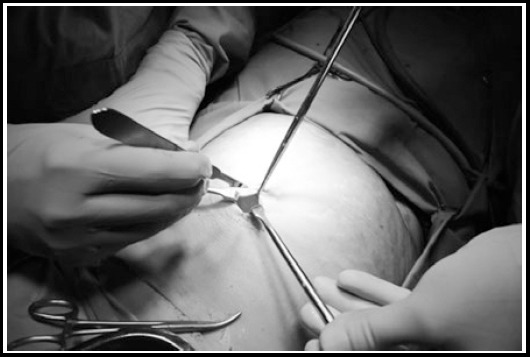
A 3 cm supra-umbilical incision made for the procedure.

**Fig.2:**
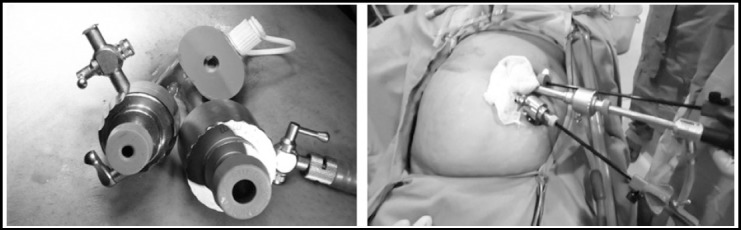
The conventional instruments passed through single supra-umbilical incision, crowding of instruments can be seen.

## DISCUSSION

SILC is an easily learnable and performable procedure which is evident from the fact that; during final phase of current study; in 05 cases, procedure was performed by junior consultant under direct supervision of senior. The reason being that the principles are essentially the same as for laparoscopic surgery with few modifications and acceptable compromises.^8^

The most work done on SILC procedures reports the use of different ports, such as triport, airseal port and X-cone with three or four holes and curved instruments. All these costly ports are for once use only making cost a major limiting factor and therefore SILC has not yet been adopted for regular use.^9^,^10^ There are very few reports where SILC has been performed identical to our study with conventional ports and instruments.

The results of the current study showed that Laparoscopic Cholecystectomy can be done using conventional instruments through a single incision, but the operating surgeon must keep in mind the different technical difficulties we encountered and same has been reported in previous studies on the same subjects.^11^-^13^ During the procedure the “laparoscope and the conventional surgical instruments are introduced through the same incision, therefore these all are on the same axis, resulting in the loss of principle of triangulation, consequently the operator and the assistant can impede the movement of each other”. Thus the different tasks during procedure like suturing, dissection, traction while performing SILC with conventional instruments is technically more demanding and in fact, not only the surgeon but also the camera assistant must be familiar with this technique. This problem of triangulation has been addressed by the use of either articulated or pre-bend instruments. However, their use requires additional learning curve. Most surgeons including us; due to the familiarity with straight instruments; preferred to use these straight instruments and find it convenient to face technical difficulties as when these arise instead to becoming familiar first with angulated instrument and then performing SILC. The clash of instruments can be avoided with the use of longer instruments, use of different size instruments and the use of 5-mm 30-degree Endo EYE (Olympus), with the CCD chip at the tip of the camera as advocated by many surgeons.^14^,^15^ In our study, we used conventional instruments because these new instruments were costly and would have an economic impact on this procedure making idea of the economy somewhat blurred.

Another key feature of SILC is the placement of retraction suture that is not usually performed in multiple port laparoscopic cholecystectomies. We find it helpful for a clear exposure of the Calot’s triangle. We employed this step in all our cases. Other studies described a “double stitch” technique for Calot’s triangle exposure; the second stitch was employed for retraction of the infundibulum.

A constant effort of surgeons to minimize the surgical trauma of multiple incisions and ports has led to the invention of SILC. Reducing the port incisions from three or four to one reduces the trauma of access and morbidity. Several studies comparing SILC with four Incisions Laparoscopic Cholecystectomy (4ILC) have been reported since 2010 and the results vary with regard to patient postoperative pain and recovery times.^16^,^17^ Report showed that in single incision laparoscopic cholecystectomy, using only the periumbilical port incision reduces the level of pain engendered by traditional multiport laparoscopic surgery.^18^ This was also shown in our study where pain is minimal after SILC with mean of 3 (Range 2-8) at visual analogue score of 1-10.

The other advantages of SILC reported in literature like better cosmesis, lowered wound complications and reduced bleeding were confirmed in our experience.

The operating time was longer in our study when compared to 4ILC however it is almost similar to many published series on SILC. The mean operating time is in fact one of the most important factor in discussion about SILC.^19^,^20^ The probable explanation for a long operating time includes; a more complicated access to the abdominal cavity and difficult handling of the instruments. But studies with large number of patients reported a comparatively reduced operating time, the reason might be the improved learning curve for this technique. This fact is evident after critical analysis of the operating time for this study that showed we spend comparatively more time during first half of this study for our initial cases when compared to cases done in later half of the study. In the cases from the first half of SILC group, the operating time was longer than those in the second half of SILC group. Another important point of discussion about SILC is conversion rate to multiple ports. In our study we converted 4(8%) patients, which is not high as compared to other published data.^17^,^19^ In contrast to the other studies on SILC, where complication rate is reported between 0% to 5%,^18^,^20^ we did not face any post-operative complications, which may be due to the fact that we have taken more time to accomplish procedure and therefore mean operating time for this series was high.

## CONCLUSION

Single incision laparoscopic cholecystectomy is safe & feasible with conventional laparoscopic instruments without additional cost of single port and articulated instruments. The cosmetic results are excellent with minimal increase in the operating time.

### Author`s Contribution

**ARS** supervision and final review.

**SAA** Manuscript writing Results compilation & interpretation.

**AM, AAS** data Collection and help in literature search & references.
